# Calix[4]amido crown functionalized visible sensors for cyanide and iodide anions†

**DOI:** 10.1039/d1ra03608d

**Published:** 2021-08-04

**Authors:** Pragati R. Sharma, Shubham Pandey, Apoorva Malik, Ganpat Choudhary, Vineet K. Soni, Rakesh K. Sharma

**Affiliations:** Department of Chemistry, Sustainable Materials and Catalysis Research Laboratory (SMCRL), Indian Institute of Technology Jodhpur NH 65, Karwar Jodhpur 342037 India rks@iitj.ac.in pragati@iitj.ac.in

## Abstract

This study comprises the design and development of calix[4] arene-amido-based ionophore*s* by varying structural stringency and steric hindrance at the lower rim to probe the anion sensing properties. The ionophores are prepared, purified, and characterized using various analytical techniques. The molecular structure of the most active ionophore I is established by single-crystal X-ray characterisation. Out of various anions investigated, iodide and cyanide show the highest sensitivity towards the ionophores investigated. Both anions are sensitive enough to give a visibly distinct color change. The binding properties of the ionophores are established with ^1^H & ^127^I NMR, fluorescence, and UV-vis spectroscopy, revealing that three ionophores strongly interact with CN^−^ and I^−^. The binding constants are calculated *via* Benesi–Hildebrand plots using absorption data. The time-dependent ^1^H NMR revealed strong hydrogen bonding between the OH and NH groups of the ionophore and cyanide anion. The ^127^I NMR shows the highest 27.6 ppm shift after 6 h for ionophore I. The crystal structure revealed hydrogen bonding of N–H protons of the amide pendulum and phenolic oxygen of the calix rim. The Job's plot depicted the possibility of a 1 : 1 complex of ionophores with both anions.

## Abstract 2

The benzyl and furfuryl functionalized calix[4] arene amido crown ionophores with structural stringency and steric hindrance showed selective colorimetric sensing towards iodide (I^−^) and cyanide (CN^−^) ions.

## Graphical abstract

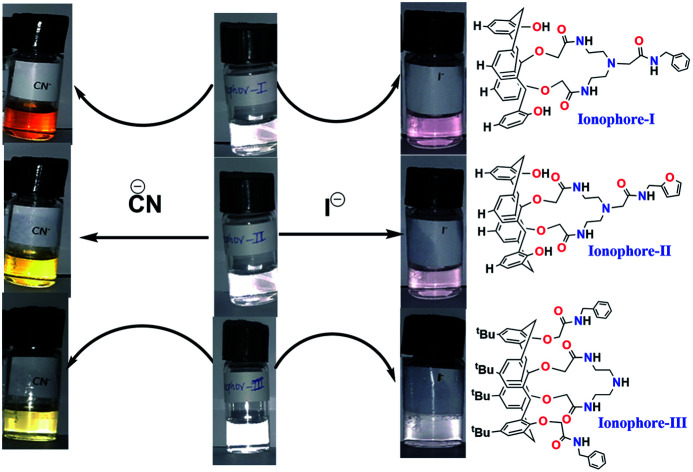

## Introduction

Supramolecular based anion-selective visual sensors have drawn significant attention in the past two decades^1^ due to their vital role in the environment and biological systems. The detection of anions is more challenging than cations due to their bigger size, structural specificity, basicity, and nucleophilicity^2^. Among various water-soluble anions, biologically important cyanide (CN^−^) and iodide (I^−^) are of great interest. Cyanide is highly toxic because of its deleterious effects on human health in minute quantities. Cyanide is also involved in chemicals, polymers, metal mining industries, and chemical warfare agents.^3^ As per the WHO guidelines, the maximum allowed cyanide concentration in drinking water is two ppb per liter.^4,5^

In contrast, iodine is an essential micronutrient for the human body, responsible for producing thyroid hormones. Iodine deficiency can also cause enlarged thyroid glands, thyroid cancer, and pregnancy complications for babies. Excessive ingestion can lead to hyperthyroidism diseases like Graves' disease.^6^ Thus, there is a dire need to develop easy, quick, and selective sensors in diverse samples.^7^ Despite the fact that several instrumental methods have been developed for the detection of cyanide and iodide ions, they are time-consuming, expensive, and cumbersome due to portability, calibration, and sample preparation. Hence there is a great need to develop colorimetric chemosensors for anions like cyanide and iodide, which are low cost and nontoxic and can be used with ease. Recently impressive advances have been made towards the development of visible cyanide sensors introducing molecules and materials such as pillararene,^8^ naphthofuran carbohydrazide,^9^ supramolecular gel,^10^ naphthalene,^11^ cyclodextrin,^12^ curcumin^13^ chromone,^14^ pyrene-thiacalix[4]arenes,^15^ calix[4]arene–naphthalimide,^16^ calix[3]pyrrole,^17^ Schiff bases,^18^ nitrobenzoxadiazole,^19^ and organogelators.^20^ For iodide detection, gold nanoparticles,^21^ poly(vinylpyrrolidone)-supported copper nanoclusters,^22^ bidentate ureido-dihomooxacalix[4]arene,^23^ hexahomotrioxacalix[3]arene,^24^ and porous ionic polymer^7^ based colorimetric sensors are noteworthy. Recently, we have reported dipicryl hydrazine based colorimetric sensors for selective determination of anions like fluoride, acetate, hydroxide, cyanide, and hydrogen sulfate. The anion binding ability of dipicrylhydrazine with various anions like fluoride, acetate, hydroxide, cyanide, and hydrogen sulfate is due to polar non-polar interactions and hydrogen bonding.^25^ Continuing our efforts, hereby we are reporting furfuryl and benzyl functionalized calix[4]amido crown-based visual molecular sensors for cyanide and iodide ions with ppm level detection limit. Limited studies pertain to the application of calix[4]amido crowns have been explored for the detection of transition metal cations^26^ and limited anions.^27^ The amide moiety of calix[4]amido crowns have a unique role in sensory behavior since N–H can interact with anions *via* selective hydrogen bonding donor.^28–31^ The furfuryl and benzyl substituent attach with amide moiety provide secondary interactions with chromophoric assistance, essential for the colorimetric sensor. These studies are unexplored in the context of cyanide and iodide. The current study also throws the light on detailed synthesis of furfuryl and benzyl functionalized calix[4]amido crowns, their stereochemistry, rigidity, steric hindrance, and interaction behavior using diverse analytical techniques such as NMR, XRD, and UV-vis.

## Experimental section

### Experimental materials

All reactions and manipulations were routinely performed under an inert atmosphere. Solvents such as toluene, ethanol, tetrahydrofuran, acetonitrile were purchased from Merck and purified by standard procedures, and freshly distilled before use. Reagents such as *p-tert*-butyl phenol and diphenyl ether, ethyl bromoacetate, chloroacetylchloride, and diethylenetriamine were purchased from Sigma-Aldrich and used as received. The furfuryl amine, benzylamine, and all tetrabutylammonium salts of anions (F^−^, Cl^−^, Br^−^, I^−^, CN^−^, OH^−^, CH_3_COO^−^, NO_3_^−^, ClO_4_^−^ and HSO_4_^−^) were purchased from Alfa-Aesar. Analytical thin-layer chromatography was carried out on silica plates (SiO_2,Merck_ 60 F_254_) Obtained from E. Merck Chemical Co.

### Physical measurement

UV-vis spectra were recorded using a Varian model Cary Win 400 UV/Vis spectrophotometer. The Perkin Elmer LS55 fluorescence spectrophotometer was used for recording fluorescence spectra. Nuclear magnetic resonance spectra (^1^H NMR & ^13^C NMR) were recorded on a Bruker 500 MHz WB FT-NMR spectrometer having proton noise decoupling mode with a standard 5 mm probe. Chemical shifts for ^1^H NMR spectra are reported as *δ* in parts per million (ppm) downfield from SiMe_4_ (*δ* 0.0) and relative to the signal of chloroform-d (*δ* 7.26, singlet). Multiplicities were given as: s (singlet); d (doublet); t (triplet); q (quartet) or m (multiplets). For proving strong binding between ionophore hosts and anionic guests, ^127^I NMR was recorded separately at 100 MHz under same solvent mixture ratio. The melting point was checked by Buchi melting point apparatus M-565. Mass spectra were recorded by Bruker microTOF-Q II using ESI source, and elemental analysis was done on FLASH EA 1112 series from Thermo finnigan, Italy.

### Synthesis and characterization of ionophores

The *p-tert*-butylcalix[4]aene, calix[4]arene, 1,3 diester calix[4]arene and calix[4]amido crown-5 were prepared as per the reported literature procedure.^26*a*,32–34^*N*-Furfurylchloroacetamide and *N*-benzylchloroacetamide reagents were synthesized according to reported methods.^35,36^

### Synthesis of ionophore I

**Scheme 1:**
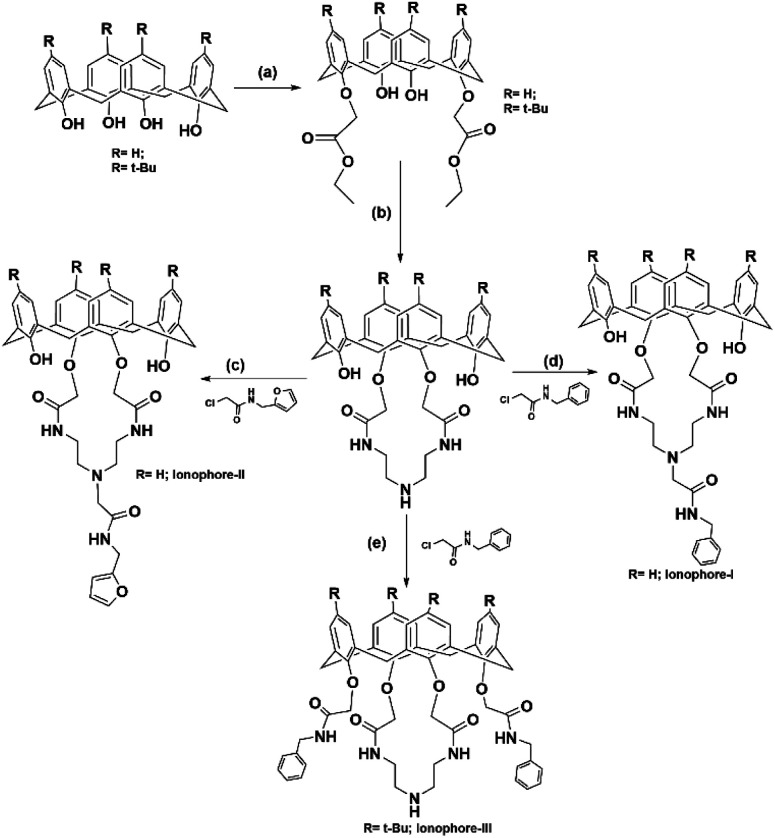
Showing synthetic procedure for compounds: (a) K_2_CO_3_, BrCH_2_COOC_2_H_5_, acetone; (b) toluene/ethanol, diethylenetriamine; (c and d) K_2_CO_3_, acetonitrile, 48 h, reflux (e) NaH, THF: DMF: 2.5 : 1, 72 h reflux.

To a solution of calix[4]amidocrown-5 (0.91 g, 1.5 mmol) in dry CH_3_CN (70 mL) were added *N*-benzyl chloroacetamide (0.83 g, 4.5 mmol) and K_2_CO_3_ (1.04 g, 7.5 mmol). The reaction mixture was refluxed for 48 h. The reaction mixture was quenched by addition of 5% HCl (10 mL) and CH_2_Cl_2_ (40 mL). The organic phase was separated and washed with water (3 × 40 mL), dried over anhydrous sodium sulphate, and evaporated *in vacuo*. The crude product was recrystallized with CHCl_3_ and methanol to yield a white crystalline compound (0.89 g, 78.95%) as shown in Scheme 1. mp 229 °C, ^1^H-NMR: (500 MHz, CDCl_3_ see ESI Fig. S1†) *δ* 8.82 (t, 2H, CONH), 7.80 (s, 2H, OH), 7.64 (t, 1H, NH), 7.37–7.31 (m, 5H, benzyl), 7.13 (d, 4H, ArH), 6.95 (d, 4H, ArH), 6.85 (t, 2H, ArH), 6.78 (t, 2H, ArH), 4.48 (d, 2H, benzyl), 4.40 (s, 4H, OCH_2_), 4.08 (d, 4H, ArCH_2_Ar AB system), 3.53 (br s, 4H, CONHCH_2_), 3.48 (d, ArCH_2_Ar, AB system), 3.26 (s, 2H, benzyl), 2.93 (br, s, 4H, CONHCH_2_CH_2_). ^13^CNMR: (see ESI Fig. S2†); *δ* 71.1, 168.7, 151.9, 150.9, 132.5, 129.7, 129.2, 129.1, 128.8, 128.6, 128.5, 127.9, 127.7, 127.6, 127.2, 126.9, 126.7, 120.9 (ArCH, ArC, CONH), 74.8 (OCH_2_), 59.9, 54.9 (benzylC), 44.19, 43.2, 39.9, 37.1, 31.9, 31.5 (CONHCH_2_CH_2_, ArCH_2_Ar), 30.3, 30.1, 29.7, 29.4, 27.1, 22.7, 19.8, 14.1 (benzylC). Maldi TOF MS ES+ (*m*/*z*) calculated for C_45_H_46_O_7_N_4_, 754.34, found 755.34 [M + H^+^]^+^, 756.34 [M + 2H^+^]^+^ (see ESI Fig. S14†) CHN Anal. calcd for C_45_H_46_O_7_N_4_: C 71.60, H 6.14, N 7.42, O 14.84; found: C 71.59, H 6.12, N 7.41, O 14.82.

### Synthesis of ionophore II

To a solution of calix[4]amidocrown-5 (0.91 g, 1.5 mmol) in dry CH_3_CN (70 mL) were added *N*-furfurylchloroacetamide (0.78 g, 4.5 mmol) and K_2_CO_3_ (1.04 g, 7.5 mmol). The reaction mixture was refluxed for 48 h. The solvent was removed *in vacuo* and the residue was quenched by addition of 5% HCl (10 mL) and CH_2_Cl_2_ (40 mL). The organic phase was separated and washed with water (3 × 40 mL), dried over anhydrous sodium sulphate, and evaporated *in vacuo*. The crude product recrystallized with CHCl_3_ and methanol to yield a white crystalline compound (0.80 g, 72.8%) as shown in Scheme 1. mp 258 °C; ^1^H NMR: (500 MHz, CDCl_3_ see ESI Fig. S3†); *δ* 8.76 (t, 2H, CONH), 7.79 (s, 2H, OH), 7.54 (t, 1H, NH), 7.12 (m, 1H, furfuryl) 7.07–7.05 (d, 4H, ArH), 6.90–6.88 (d, 4H, ArH), 6.78 (t, 2H, ArH), 6.71 (t, 2H, ArH), 6.18 (s, 2H, furfuryl), 4.45 (s, 4H, OCH_2_), 4.39 (d, 2H, CH_2_ furfuryl), 4.09 (d, 4H, ArCH_2_Ar, AB system), 3.47 (d, 4H, ArCH_2_Ar, AB system), 3.42 (d, 4H, CH_2_), 3.18 (br s, 2H, CONHCH_2_), 2.82 (brs, 4H, CONHCH_2_CH_2_). ^13^CNMR: (see ESI Fig. S4†): *δ* 171.1, 168.7, 152.2, 150.9, 141.9, 132.5, 129.7, 129.2, 127.6, 126.7, 120.9 (ArCH, ArC, CONH), 110.5, 106.8 (furfuryl C), 74.9 (OCH_2_), 59.61, 54.92 (furfuryl C), 39.9, 36.3, 31.9, 31.5 (CONHCH_2_CH_2_, ArCH_2_Ar), 29.7, 22.70, 14.1 (furfuryl C). Maldi TOF MS ES+ (*m*/*z*) calculated for C_43_H_44_O_8_N_4_, 744.32, found 745.32 [M + H^+^]^+^, 746.32 [M + 2H^+^]^+^, 747.32 [M + 3H^+^]^+^ (see ESI Fig. S15†), CHN Anal. calcd for C_43_H_44_O_8_N_4_: C 69.34, H 5.95, N 7.52, O 17.18; found: C 69.30, H 5.91 N 7.41, O 17.10.

### Synthesis of ionophore III

To a solution of *p-tert*-butyl calix[4]amido crown-5 (1.24 g, 1.5 mmol) in dry THF (50 mL) and dry DMF (20 mL) under N_2_ was added NaH (0.18 g, 7.5 mmol). The mixture was stirred at RT for 30 min followed by addition of *N*-benzyl chloroacetamide (0.83 g, 4.5 mmol). The reaction mixture was refluxed for 72 h, the solvent was removed *in vacuo* and quenched by the addition of 5% HCl (10 mL) and CH_2_Cl_2_ (40 mL). The organic phase was separated and washed with water (3 × 40 mL), dried over anhydrous sodium sulfate and evaporated *in vacuo*. The crude product recrystallized with CHCl_3_ and methanol to yield a white crystalline compound (1.126 g, 67.42%) as shown in Scheme 1. mp 244 °C; ^1^H-NMR: (500 MHz, CDCl_3_ see ESI Fig. S5†); *δ* 8.82 (t, 2H, CONH), 7.66 (t, 1H, CONH), 7.41 (s, 2H, ArH), 7.28 (m, 6H, benzyl), 7.20 (m, 4H, ArH), 7.11 (s, 4H, ArH), 6.85 (s, 4H, ArH), 4.47 (d, 2H, benzyl), 4.38 (s, 4H, OCH_2_), 4.04 (d, 4H, ArCH_2_Ar AB system), 3.51 (br s, 4H, CONHCH_2_), 3.45 (s, 4H, CONHCH_2_), 3.40–3.37 (d, ArCH_2_Ar, AB system), 3.26 (s, 2H, benzyl), 2.91 (br, s, 4H, CONHCH_2_CH_2_), 1.63 (1H, NH), 1.30 (s, 18H, *t*-butyl), 0.99 (s, 18H, *t*-butyl). ^13^CNMR: (see ESI Fig. S6†); *δ* 170.1, 167.9, 148.4, 147.6, 147.5, 142.53, 137.86, 130.9, 127.5, 126.5, 126.4, 126.1, 125.1, 124.6 (ArCH, ArC, CONH), 73.6 (OCH_2_), 58.7, 53.9 (benzylC), 42.16, 38.5, 37.1, 33.1, 32.9, (CONHCH_2_CH_2_, ArCH_2_Ar), 30.7, 30.5, 29.8, 29.7, 28.6, 21.8, 13.1 (benzyl C). Maldi TOF MS ES+ (*m*/*z*) calculated for C_70_H_87_O_8_N_5_, 1125.66, found 1126.66 [M + H^+^]^+^, 1127.66 [M + 2H^+^]^+^ (see ESI Fig. S16†), CHN Anal. calcd for C_70_H_87_O_8_N_5_: C 74.6, H 7.78, N 6.22, O 11.36; found: C 74.9, H 7.12, N 6.41, O 11.82.

### Visual detection experiment

**Fig. 1:**
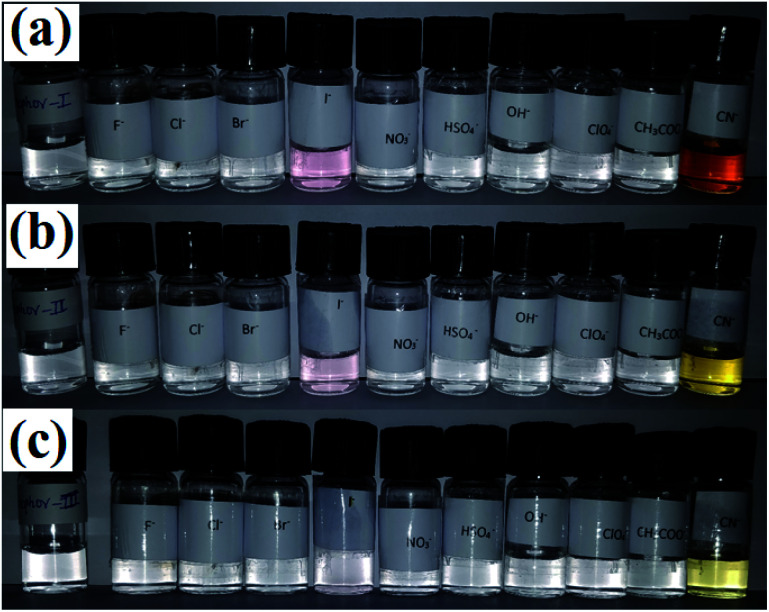
Colorimetric analysis for (a) ionophore I, (b) ionophore II, and (c) ionophore III upon addition of different anions.

Visual detection experiments were carried out for recognizing the host–guest interactions of ionophores I–III with various anions. In a typical experiment, a series of stock solutions of anions (5 × 10^−3^ M) and ionophores (5 × 10^−5^ M) were prepared in freshly distilled acetonitrile. Further, 2 mL aliquots of stock solution were taken and mixed with 2 mL of the anion solution. Typical photographs were taken with a digital camera. All ionophores gave instant color change for iodide and cyanide ions. Iodide and cyanide ion gave pink and wine color for ionophore I, respectively, consistent with the color variation displayed in Fig. 1. The ionophore II showed light pink color with iodide while cyanide turned yellow. In contrast to ionophore I and II, ionophore III gives light yellow color with cyanide, but there is no visible change in the case of iodide.

### Interaction with anions by UV-vis study

In UV-vis titration experiment, 1 mL (5 × 10^−3^ M) stock solution of tetrabutylammoniumanions (F^−^, Cl^−^, Br^−^, I^−^, CN^−^, OH^−^, CH_3_COO^−^, NO_3_^−^, ClO_4_^−^ and HSO_4_^−^) was added to a 1 mL (5 × 10^−5^ M) acetonitrile solution of ionophore I–III in ascending order in ten factions (1 : 100 ratio). The spectral changes were recorded to ascertain the interaction/selectivity. Based on spectral changes, titrations were performed with strongly interacted anions cyanide and iodide to investigate the binding sites and determine the binding constant. We have performed competitive binding with other anions (F^−^, Cl^−^, Br^−^, I^−^, CN^−^, OH^−^, CH_3_COO^−^, NO_3_^−^, ClO_4_^−^ and HSO_4_^−^) and found that ionophores are showing selective binding and color change towards cyanide.

### NMR spectral study

The ^1^H and ^13^C NMR spectra of ionophore I–III were recorded before and after the addition of selected anions (CN^−^ and I^−^) in CDCl_3_ : CD_3_CN (1 : 2.5). In this experiment, 0.003 mmol of ionophore I–III were dissolved separately in 0.5 mL mixture containing CDCl_3_ : CD_3_CN (1 : 2.5), and further, add various anions (20 molar equivalents) in the form of TBA salts. Proton NMR was recorded at different time intervals up to 24 h after the addition of anions.

### Structure determination by single-crystal X-ray study

Appropriate crystal was selected for ionophore I, dipped in paratone oil, and mounted on cryo loop. Crystal data were collected at 100 K using graphite monochromatic MoKα (*λ* = 0.71073 Å) radiation on a Bruker SMART APEX diffractometer equipped CCD area detector. The SAINT software^37^ was used for data integration and reduction. Empirical absorption correction was applied to the collected reflections with SADABS.^38^ SHELXTL^39^ was used to solve the structures by direct methods using and refined on *F*_2_ by the full-matrix least-squares technique using the SHELXL-97 (ref. 40) package. Non-hydrogen atoms were refined anisotropically till convergence is reached. The MERCURY 3.8 (ref. 41) is used to generate graphics. The X-ray structure of ionophore I is shown in Fig. S17,† wherein chloroform and water molecules have been omitted for clarity. The phenolic OH of the alternate phenyl rings of the calix moiety are substituted by amide-linked chains. The presence of hydrogen bonding between amide NHs and phenolic oxygen atoms is also observed. Crystallographic parameters for the ionophore I are given in Table S1.†

## Result and discussion

### UV-vis absorption studies

**Fig. 2:**
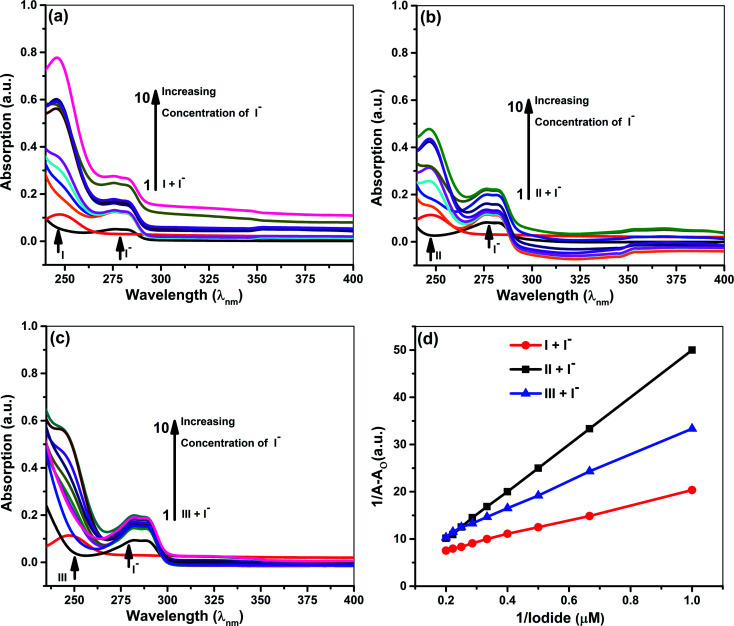
Absorption spectra of 5 × 10^−5^ M ionophores in the absence and presence of iodide at concentrations 5 × 10^−5^ to 5 × 10^−6^ M (a) ionophore I (b) ionophore II (c) ionophore III (d) Benesi–Hildebrand absorption plot for 1 : 1 complexation of iodide with ionophores I–III.

**Fig. 3:**
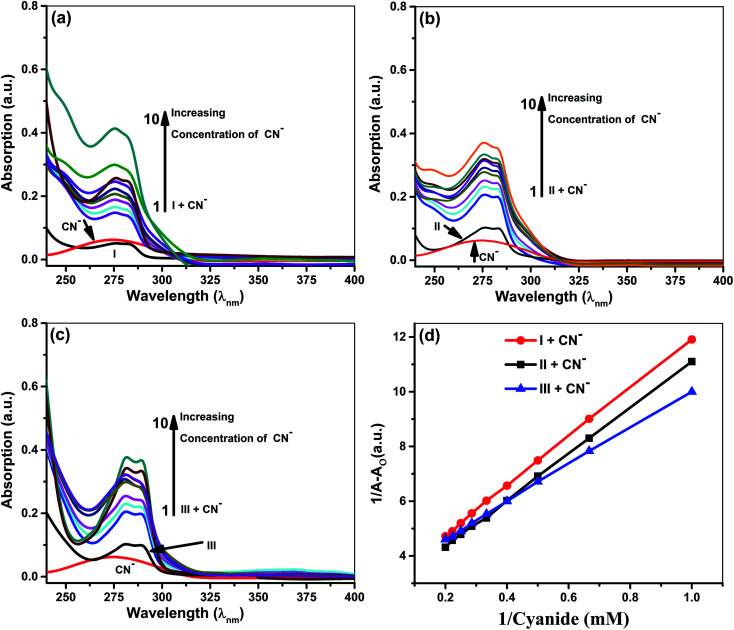
Absorption spectra of 5 × 10^−5^ M ionophore's in the absence and presence of cyanide at concentrations 5 × 10^−3^ to 5 × 10^−4^ M (a) ionophore I (b) ionophore II (c). Ionophore III (d) Benesi–Hildebrand absorption plot for 1 : 1 complexation of cyanide with ionophores I–III.

Quantitative investigations were carried out to understand cyanide and iodide anions' encapsulation behavior, in presence of different ionophores using a spectrophotometric titration method in CH_3_CN. The absorption spectra of ionophores I, II, and III at variable concentrations of iodide and cyanide are shown in Fig. 2 and 3. Ionophores I and II show a strong absorption maximum at 279 nm, while III shows at 285 nm. Single spectral band for anions and dual spectral bands were observed for all ionophores from 273 to 290 nm. Upon increasing the anions' concentration, the absorbance of ionophores is increased at both the maxima as per Fig. 2a–c (for iodide) and Fig. 3a–c (for cyanide). In the absence of ionophore, iodide and cyanide anions show their respective peaks at 249 and 275 nm, respectively. The absorbance increased with the concentration of anions due to interaction with ionophores. Changes in the absorbance of ionophores (measured against the solvent as reference) upon the addition of anions were observed at 279 nm for ionophore's I and II, and 285 nm for ionophore III. Owing to their hydrophobic cavities, host ionophores offer the iodide and cyanide a suitable environment for interaction to form inclusion complexes. The binding constants for the formation of ionophores: iodide and ionophores: cyanide complexes are determined by analyzing the absorbance changes. The binding constant *K*_a_ and stoichiometry of the inclusion complexes of ionophores with iodide and cyanide can be determined by the Benesi–Hildebrand equation (Table 1).^42^ For ionophores I, II and III, the calculated binding constant from the straight-line slope for iodide is found to be 552 M^−1^, 509 M^−1^, and 350 M^−1^, respectively. At the same time, for cyanide, the values are lower, *i.e.*, 319 M^−1^, 304 M^−1^ and 209 M^−1^, respectively, at 298 K. This behavior explains strong complexation towards both anions in the following order: I > II > III. It is observed that ionophore I gave a stronger *K*_a_ value than II and III, indicating its superior interaction compared to heavily alkylated and arylated II and III. Considering the structural features of the host and guest, we presumed that the deprotonation and charge transfer *via* polarization leads to complexation with iodide. In contrast, hydrogen bonding between cyanide and amines of ionophore leads to deprotonation and strengthens the host–guest association. All the above phenomena are common in all ionophores. Since steric hindrances are in the following order: III > II > I, bind in reverse order.

**Table 1:** Binding constants of the complexes *via* absorption spectral maxima for iodide and cyanide anions with Ionophores I, II and III

S. No.	Ionophores	*λ* (nm)	Monitored *λ*_max_ (nm)	Binding constant *K*_a_ (M^−1^)	*R* ^2^	Complex
**For iodide anion**						
1	I	273–284	279	552.0	0.9988	1 : 1
2	II	273–284	279	509.0	0.9999	1 : 1
3	III	280–290	285	350.0	0.9982	1 : 1

**For cyanide anion**						
4	I	273–284	279	319.0	0.9996	1 : 1
5	II	273–284	279	304.0	0.9996	1 : 1
6	III	280–290	285	209.0	0.9993	1 : 1

The plot's good linearity with better correlation co-efficient *R*^2^ more than 0.998 for all complexes shows the strong formation of 1 : 1 complexes between anions and ionophores I, II, and III. Stoichiometric ratios are calculated from Job's Plot, which indicates towards 1 : 1 complex formation (see ESI Fig. S7†).^43^

### Fluorescence studies

**Fig. 4:**
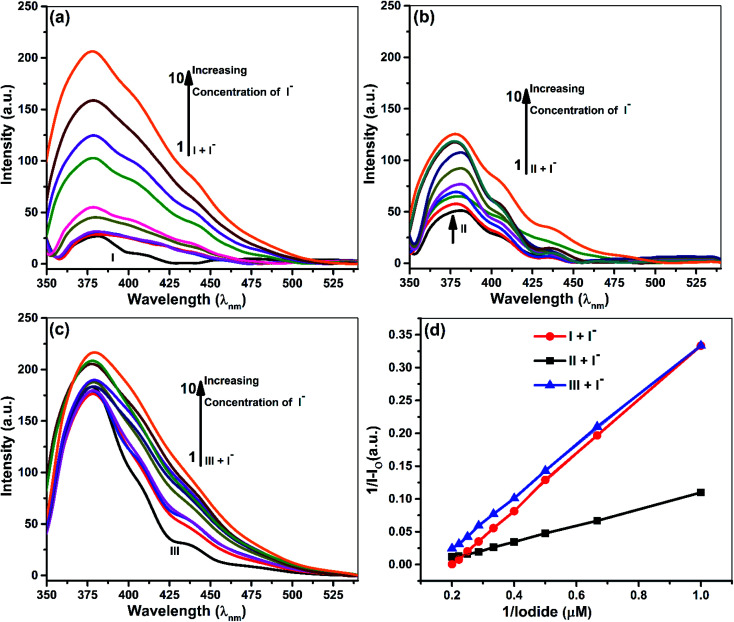
Fluorescence spectra of 5 × 10^−5^ M ionophores in the absence and presence of iodide at concentrations 5 × 10^−5^ M to 5 × 10^−6^ M. (a) Ionophore I (b) ionophore II (c) ionophore III (d) Benesi–Hildebrand emission plot for 1 : 1 complexation of iodide with ionophores I–III.

**Fig. 5:**
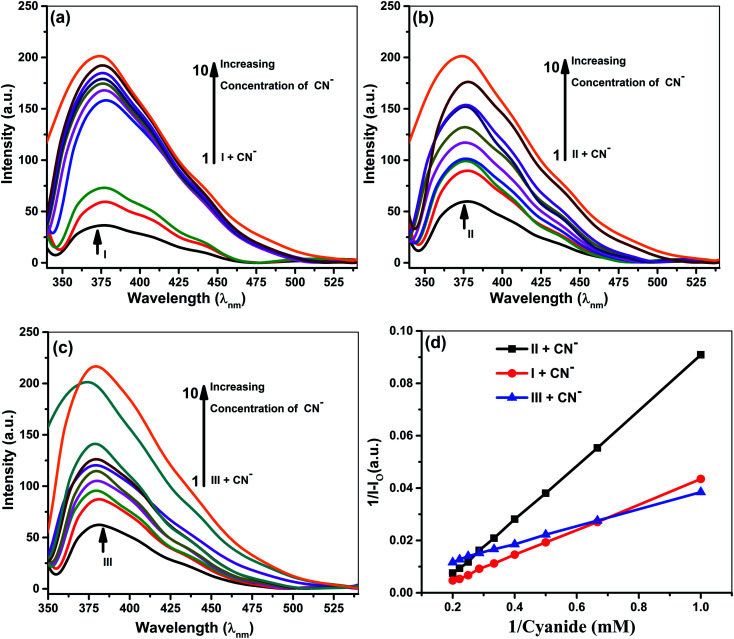
Fluorescence spectra of 5 × 10^−5^ M ionophores in the absence and presence of cyanide at concentrations 5 × 10^−3^ M to 5 × 10^−4^ M. (a) Ionophore I (b) ionophore II (c) ionophore III (d) Benesi–Hildebrand absorption plot for 1 : 1 complexation of cyanide with ionophores.

Samples prepared for UV-vis analysis were further used for the spectrofluorometric study. Table 2 and Fig. 4 & 5 show the supramolecular interaction of ionophores with iodide and cyanide anions. An enrichment of fluorescence intensity of ionophores was observed upon adding iodide and cyanide aliquots. Such qualitative assessment of the inclusion complexation behavior by spectral titrations shows a single emission maximum at 379 nm for ionophore I and II and 380 for ionophore III, respectively, with very low fluorescence intensity before the addition of anions at excitation wavelength of 279 nm for I and II and 285 nm for ionophore III. In the case of iodide anion, there is no spectral shift observed in ionophore II and III, while ionophore I shows the blue shift to 375 nm (∼5 nm). Fig. 4 and 5 shows the emission spectra of iodide and cyanide anion with all ionophores.

**Table 2:** Binding constants of the complexes *via* emission spectral maxima for iodide and cyanide anions with ionophores I, II and III

S. No.	Ionophores	*λ* (nm)	Monitored *λ*_max_ (nm)	Binding constant *K*_a_ (M^−1^)	*R* ^2^	Complex
**For iodide anion**						
1	I	375–385	379	834	0.9997	1 : 1
2	II	375–385	379	729	0.9995	1 : 1
3	III	380–384	380	498	0.9990	1 : 1

**For cyanide anion**						
4	I	375–385	379	956	0.9994	1 : 1
5	II	375–385	379	753	0.9998	1 : 1
6	III	380–384	380	604	0.9991	1 : 1

Enhancement of emission maxima shows gradual redshift for I and II from 379 nm to 375 nm, while no noticeable changes were observed for ionophore III. Alteration in the host molecule's photophysical and photochemical properties establishes transference of the anions from a more protic atmosphere to a less protic atmosphere, *i.e.*, the cavity of ionophores. The ionophores can form intra and inter-molecular hydrogen bonds between the phenolic O–H and the nitrogen of the cyanide. The supramolecular cavity of ionophores offers a protective environment with excitation of singlet species and vibrational restriction to iodide and cyanide molecules during encapsulation. The study clearly explained that the encapsulation or inclusion phenomenon depends on the fitting and selective detection concept between host and guest molecules. Such encapsulations cannot be clearly explained on single parameter, various spectacles leads to several weak intermolecular forces such as ion–dipole, dipole–dipole, van der Waals, electrostatic forces, hydrogen bonding strengths, and steric effects. Not only this, hydrogen-bonding donor binding to an anions involves deprotonation of hydrogen binding donor, in which proton transferred to basic anions and it varies with size and electronegative character of anions CN^−^ and I^−^.

All the above concepts collaborate and contribute to the formation of a stable inclusion. Hence, inclusion formation constants or binding constants (*K*_a_) of all the complexes were calculated from fluorescence data using the Benesi–Hildebrand equation, and the stoichiometric ratio was determined (Table 2).^42,43^ A good linearity with a better regression co-efficient, *R*^2^ is obtained. The binding constant ‘*K*_a_’ is calculated from the straight line's slope, considering both ground and excited state measurements. It was found to be 834 M^−1^, 729 M^−1^ & 498 M^−1^ in the case of iodide anion, while the derived binding constant from cyanide complexes were 956 M^−1^, 753 M^−1^ & 604 M^−1^ for ionophore I, II & III, respectively. The above behavior indicates that all ionophore forms different inclusion complexes with cyanide and iodide ions. The linearity of the plot shows that the stoichiometry of the complex between ionophores and both the anions was found to be 1 : 1. The binding constant values for ionophore I with all anions are higher than other complexes (Table 2), indicating that ionophore's capability to form inclusion complexes with iodide and cyanide anions. The ionophore I can complex better with the cyanide and iodide than arylated II and III. For iodide, deprotonation and charge transfer *via* polarization are major interacting forces. However, hydrogen bonding between nitrogen atoms of cyanide and amines of ionophore leads to complete deprotonation and strengthening host–guest association. All above phenomena were common in all ionophores; however, steric hindrances are highest in III, which have the least binding. Stoichiometric ratios were calculated from Job's plot that shows 1 : 1 complex formation (see ESI Fig. S7†).^43^ Binding constant data's for emission and absorption data's were different, but dual binding constants and better *R*^2^ values in same sequence clearly explain that ionophores have a strong binding capability with anions.

### ^1^H & ^127^I NMR study

**Fig. 6:**
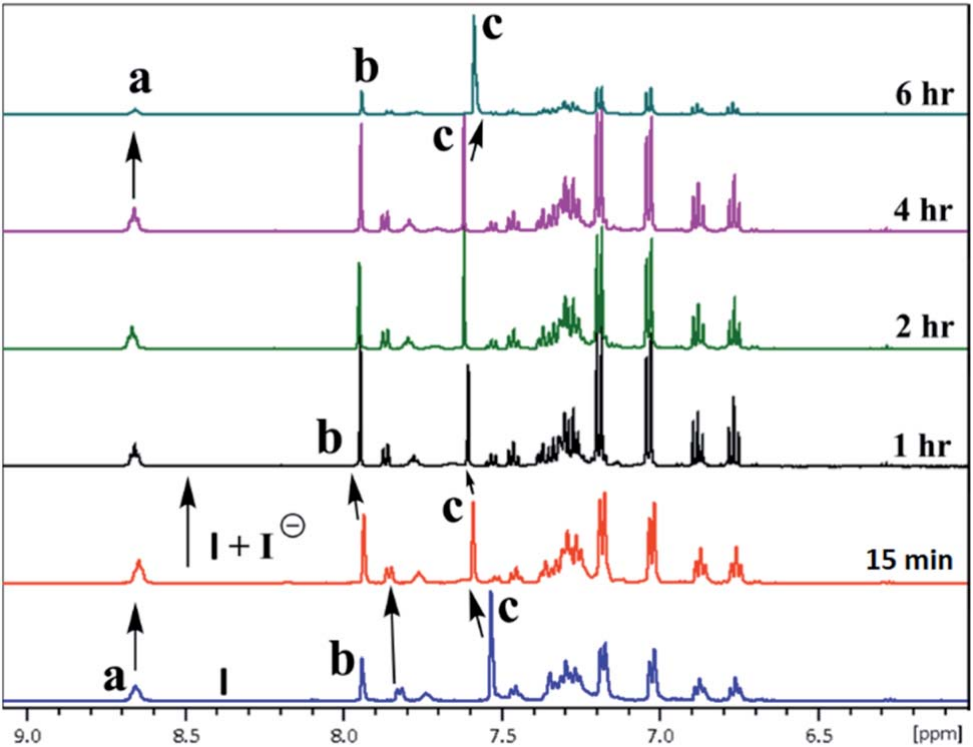
A part of time-dependent ^1^H NMR spectra (500 MHz) in CDCl_3_: CD_3_CN at 25 °C showing protons of ionophore I in the absence presence of iodide anion.

**Fig. 7:**
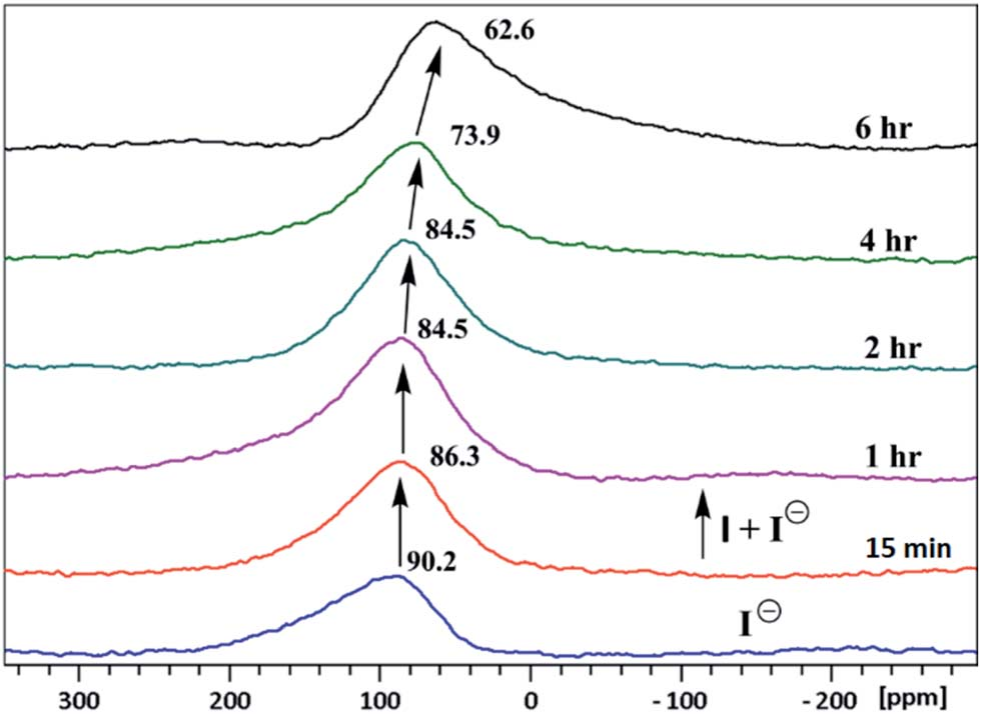
Time-dependent ^127^I NMR spectra (500 MHz) in CDCl_3_: CD_3_CN at 25 °C of tetrabutylammonium iodide with ionophore I.

The inclusion of guest ions inside the ionophore's cavity was ascertained by changes observed in ^1^H & ^127^I NMR. The NMR interaction studies were carried out by adding the equimolar concentration of ionophore I and anions at 297 K. For iodide anion there is slight significant change was seen in time-dependent ^1^HNMR (Fig. 6) due to the large size and poor hydrogen bonding tendency. Peak a get diminished with slight shift in case of peak c and becomes constant after 6 h. Interestingly, the ^127^I showed 27.6 ppm upfield shift due to inclusion and non-covalent interaction inside the cavity (Fig. 7). No further changes were observed after 6 h. The other two ionophores II and III, also showed similar trends (see ESI Fig. S8–S13†).

**Fig. 8:**
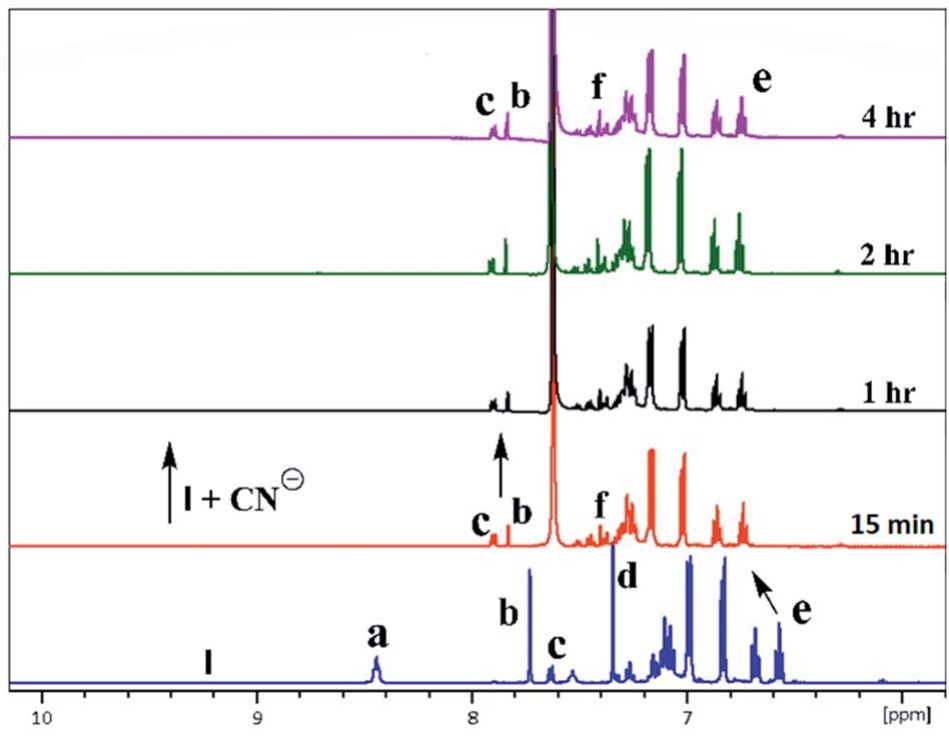
A part of time-dependent ^1^H NMR spectra (500 MHz) in CDCl_3_: CD_3_CN at 25 °C for tetrabutylammonium cyanide anion with ionophore I.

In the case of cyanide, clear changes were observed in time-dependent proton NMR with deprotonation of peak 8.45 ppm (–NH) and 7.75 ppm (–OH) of ionophores within 15 min, as shown in Fig. 8. For ionophore I, aromatic peaks shifted downfield due to the strong nucleophilicity of cyanide ion inclusion. It has been observed that ionophores do not behave chemidosmetrically as the color started fading after 72 h and the N–H at 8.45 ppm proton started reappearing (Fig. S18†). The sterically crowded ionophore III showed the least binding capability as observed in ^127^I NMR shift (see ESI Fig. S13†).^7*b*^

## Conclusions

Three new functionalized furfuryl and benzyl derivated of calix[4]amido crown based molecular ionophores were synthesized to investigate their anion sensing properties. These ionophores were designed with variation in lower rim conformation based on rigidity and steric hindrance at the binding site. A number of anions were investigated with three ionophores indicating that ionophore I bind strongly with both iodide and cyanide ions with interaction order I > II > III. The ionophore I shows drastic color change from colorless to pink and wine red for iodide and cyanide anion. For iodide ion, ionophore II and III do not show very sharp color change while CN^−^ gave colorless to light yellow. The strong binding for iodide ion was evident by ^127^I NMR where ionophore I gave 27.6 ppm upfield shift while for ionophore II and III, 13.9 and 10 ppm, respectively. The cyanide ion shows strong interaction with N–H and O–H groups, with all three ionophores, resulted in a color change. The color change is attributed to the charge transfer process among the anions and lower amide pendulum. The ionophore I interact strongly with iodide and cyanide because of the less hindered binding site, for effective interaction with anions. The binding isotherms were fitted to a 1 : 1 binding model with better binding constants, as suggested by the Benesi–Hildebrand plot for all ionophores.

## Conflicts of interest

There are no conflicts to declare.

## Supplementary Material

RA-011-D1RA03608D-s001

RA-011-D1RA03608D-s002
