# Supplementary material for: Calix[4]amido crown functionalized visible sensors for cyanide and iodide anions
Source: RSC Adv. 2021 Aug 4;11(43):26644–54. doi: 10.1039/d1ra03608d (PMC9037394; doi:10.1039/d1ra03608d)
Supplement: RA-011-D1RA03608D-s001 [file RA-011-D1RA03608D-s001.pdf]

Electronic Supplementary Material (ESI) for RSC Advances.
This journal is © The Royal Society of Chemistry 2021

Electronic Supporting Information

Calix[4]amido Crown Functionalized Visible Sensors for Cyanide and Iodide Anions

Pragati R Sharma,*[a] Shubham Pandey,[a] Apoorva Malik,[a] Ganpat Choudhary,[a] Vineet K Soni[a] and Rakesh K Sharma*[a]

Sustainable Materials and Catalysis Research Laboratory (SMCRL),
Department of Chemistry, Indian Institute of Technology Jodhpur
NH 65, Karwar, Jodhpur-342037, India

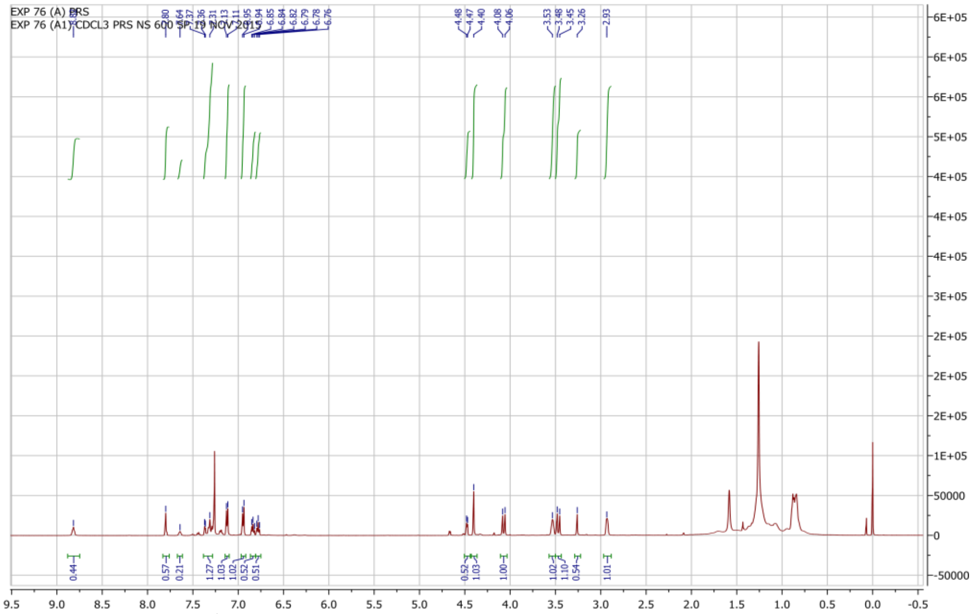

Figure S1. ^{1}HNMR spectrum of Ionophore I (500 MHz, $CDCl_3$at 25˚C).

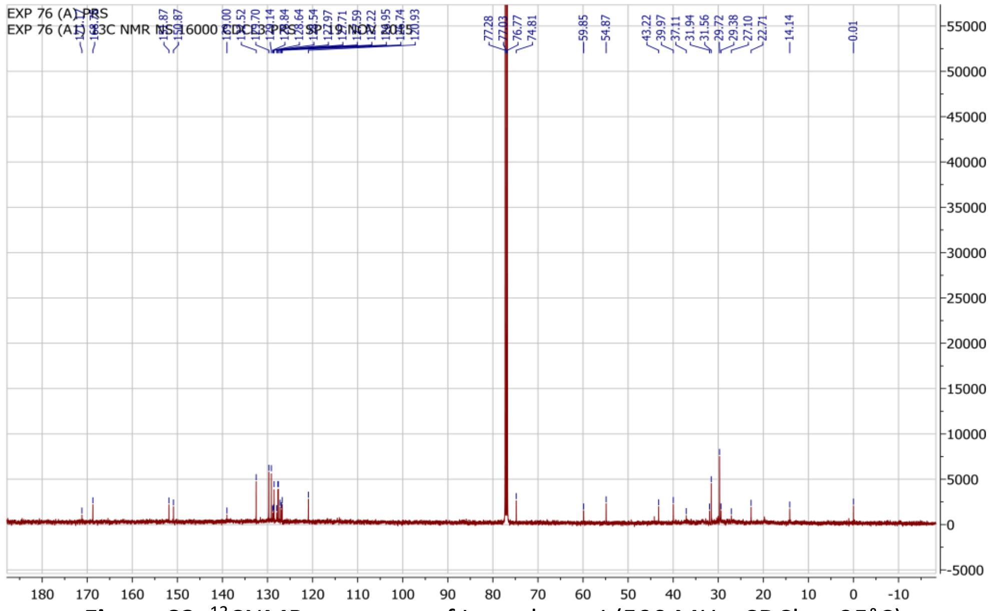

Figure S2. ^{13}CNMR spectrum of Ionophore I (500 MHz, $CDCl_3$at 25˚C).

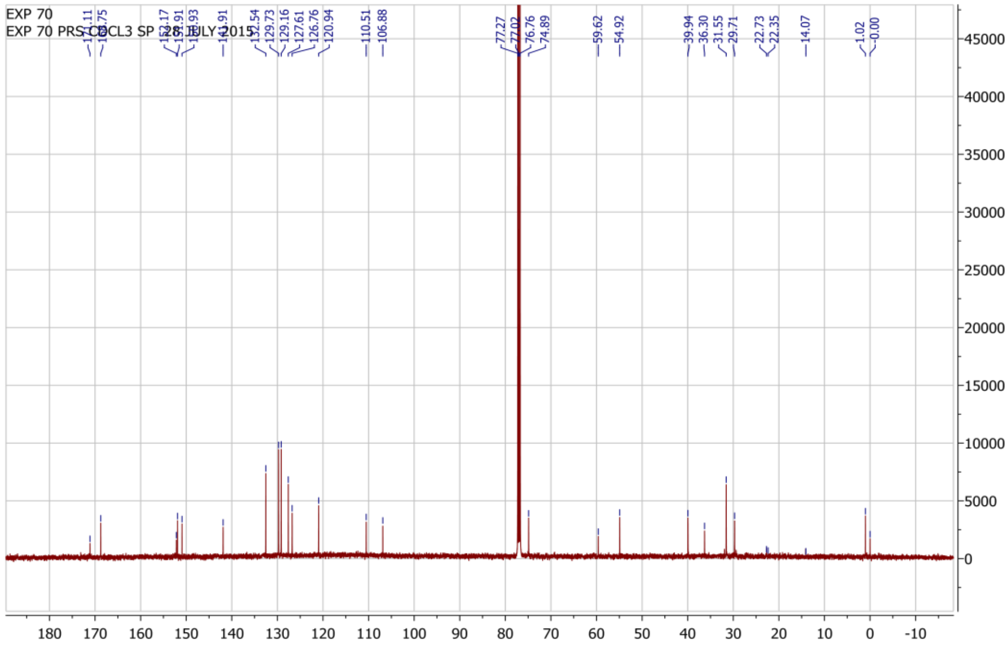

Figure S3. ^{1}HNMR spectrum of Ionophore II (500 MHz, $CDCl_3$at 25˚C).

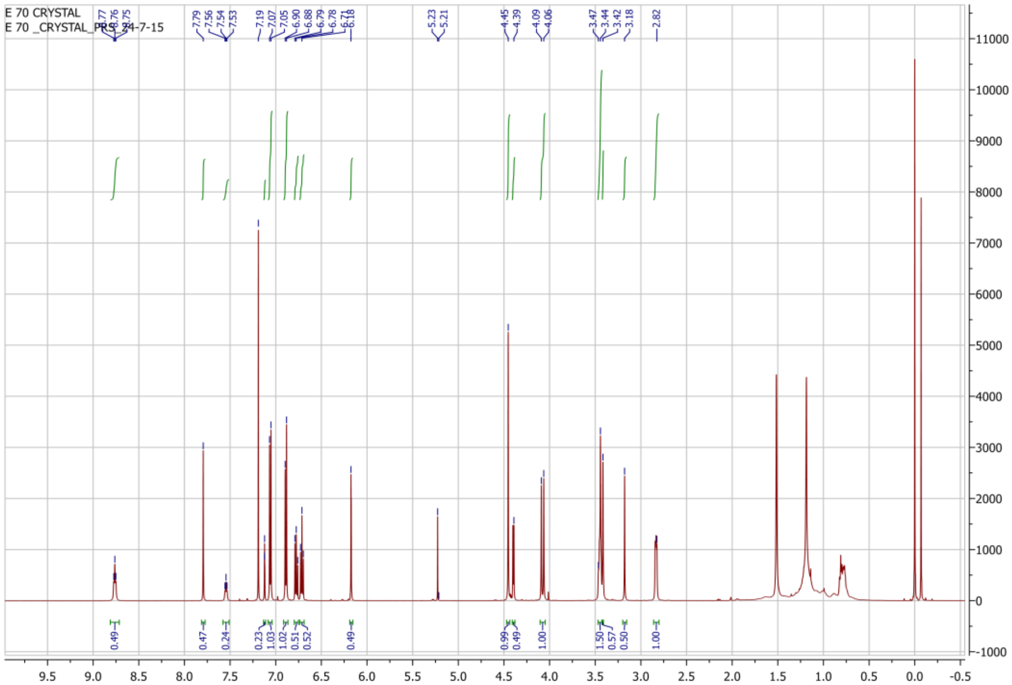

Figure S4. ^{13}CNMR spectrum of Ionophore II (500 MHz, $CDCl_3$at 25˚C).

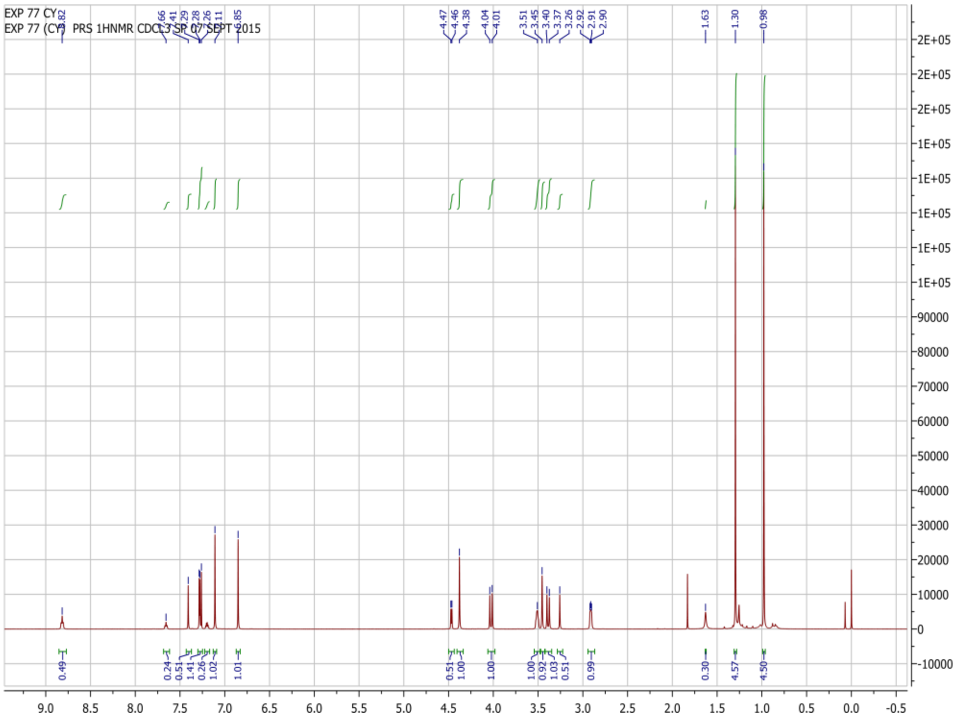

Figure S5. ^{1}HNMR spectrum of Ionophore III (500 MHz, $CDCl_3$at 25˚C).

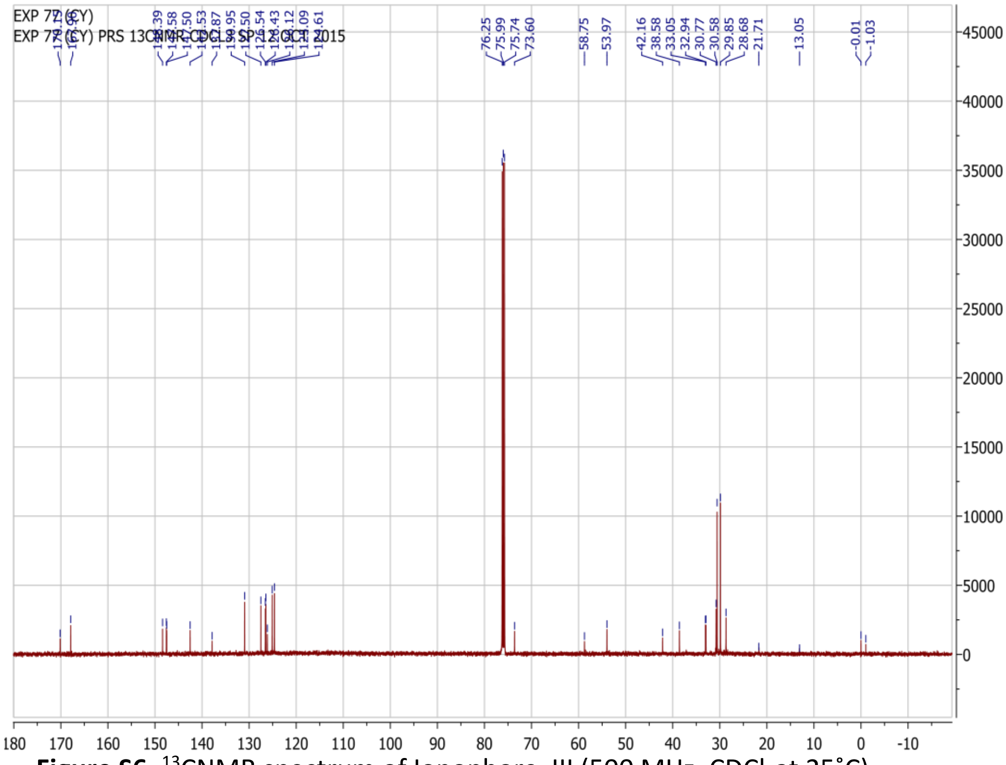

Figure S6. ^{13}CNMR spectrum of Ionophore III (500 MHz, $CDCl_3$at 25˚C).

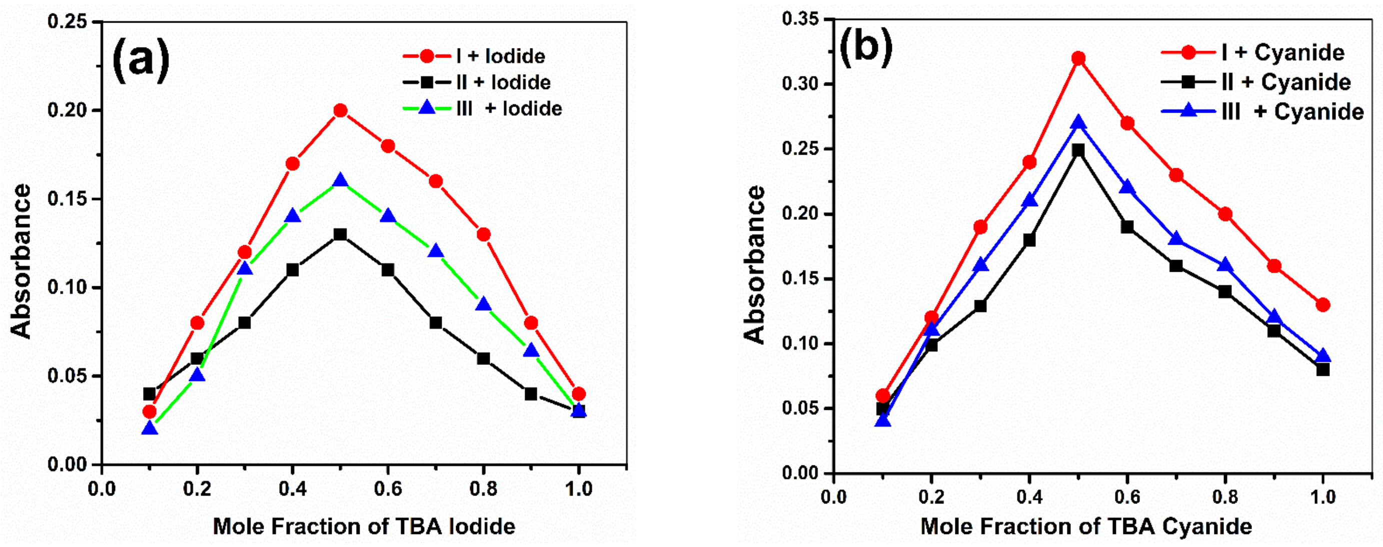

Figure S7. Showings Job's Plot for anions capture by ionophore's (a) TBA salt of iodide and (b) TBA salt of cyanide.

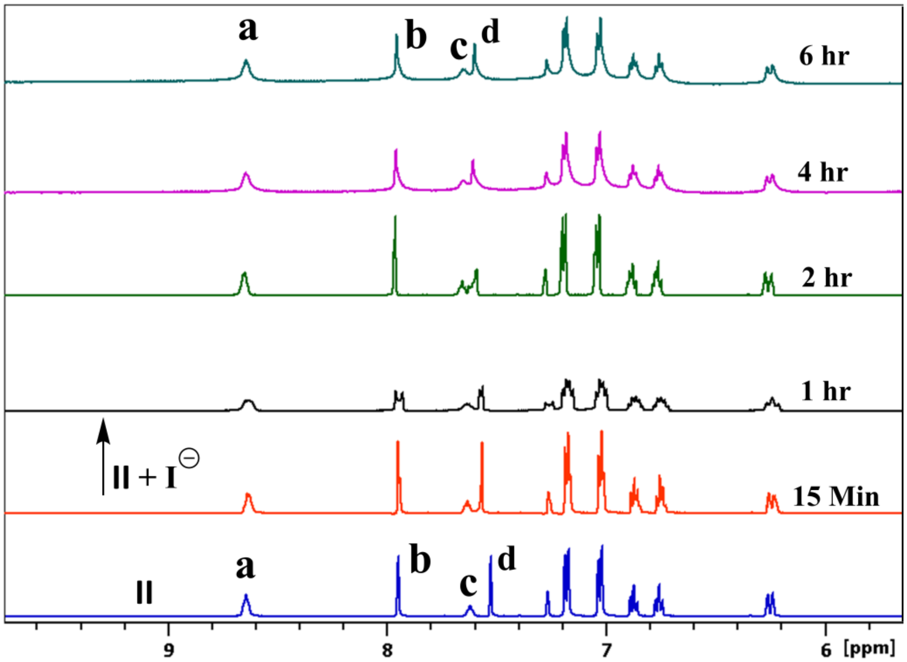

Figure S 8. A part of time-dependent ^{1}H NMR spectra (500 MHz) in $CDCl_3$:CD_3CN at 25˚C showing protons of Ionophore II in the absence and the presence of Iodide anion.

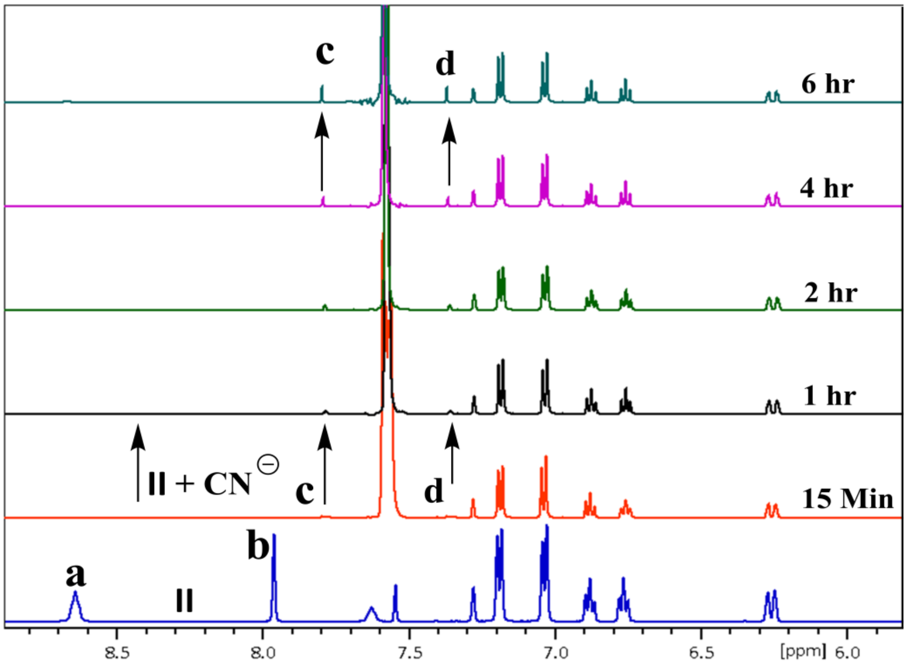

Figure S 9. A part of time-dependent ^{1}H NMR spectra (500 MHz) in $CDCl_3$:CD_3CN at 25˚C showing protons of Ionophore II in the absence and the presence of cyanide anion.

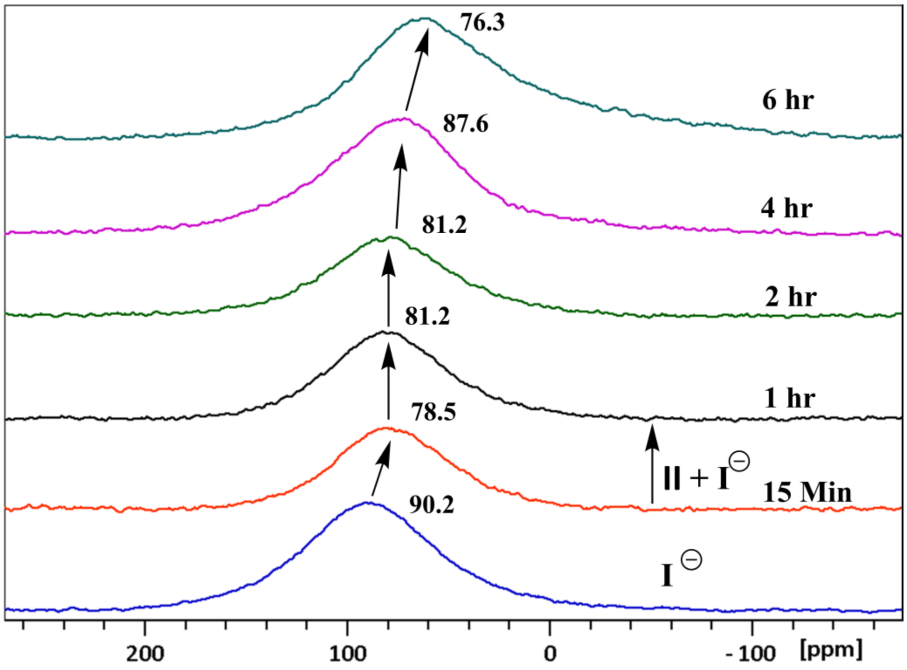

Figure S10. Time-dependent ^{127}I NMR spectra (500 MHz) in $CDCl_3$: CD_3CN at 25˚C showing ^{127}I peaks of Tetrabutylammonium Iodide the absence, as well as in the presence, of Ionophore **II**.

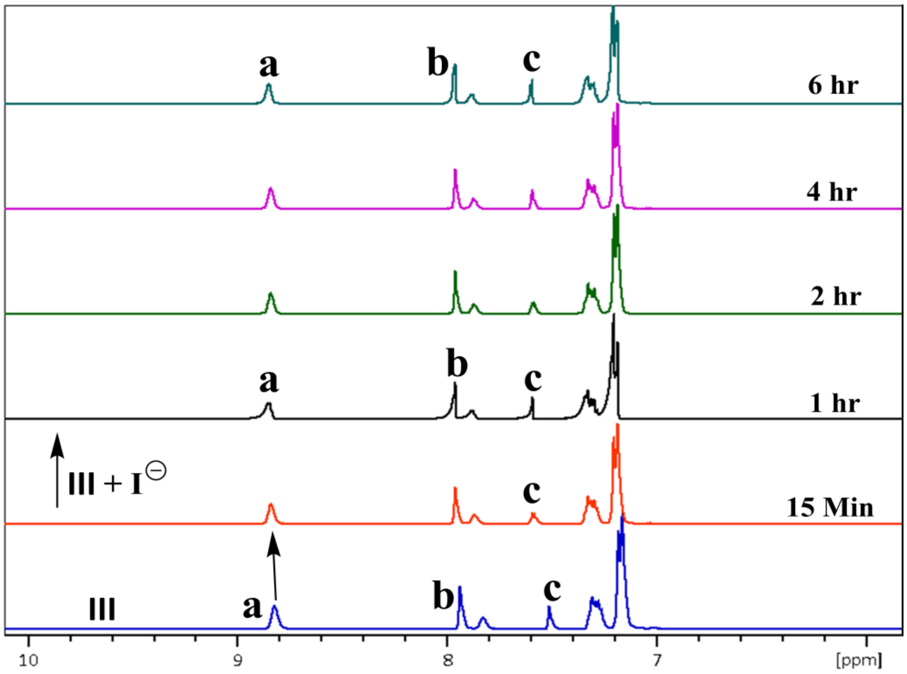

Figure S11. A part of time-dependent ^{1}H NMR spectra (500 MHz) in $CDCl_3$: CD_3CN at 25˚C showing protons of Ionophore III in the absence, as well as in the presence, of Iodide anion.

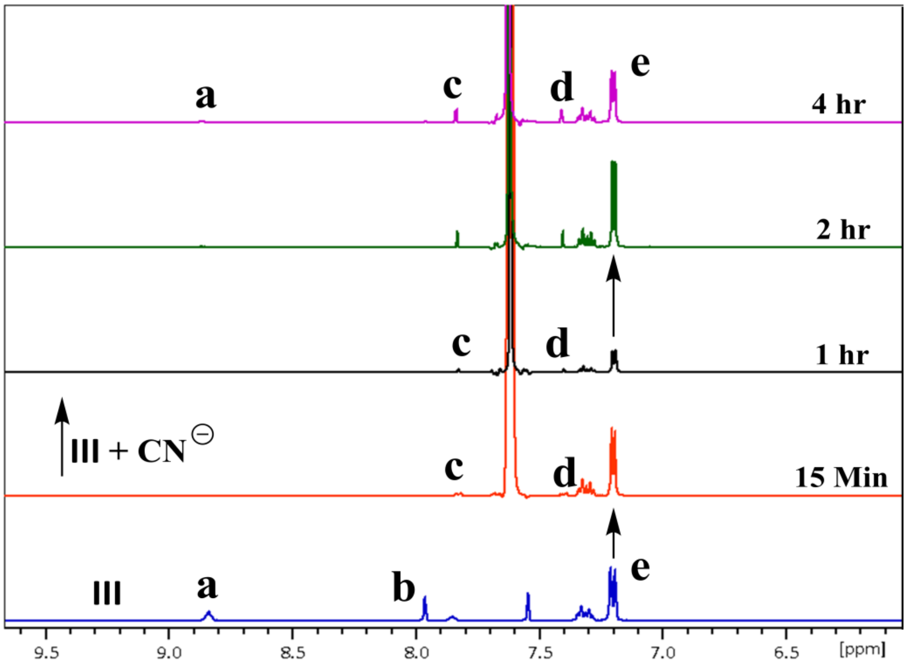

Figure S12. A part of time dependent ^{1}H NMR spectra (500 MHz) in $CDCl_3$: CD_3CN at 25˚C showing protons of Ionophore **III** in the absence, as well as in the presence, of cyanide anion.

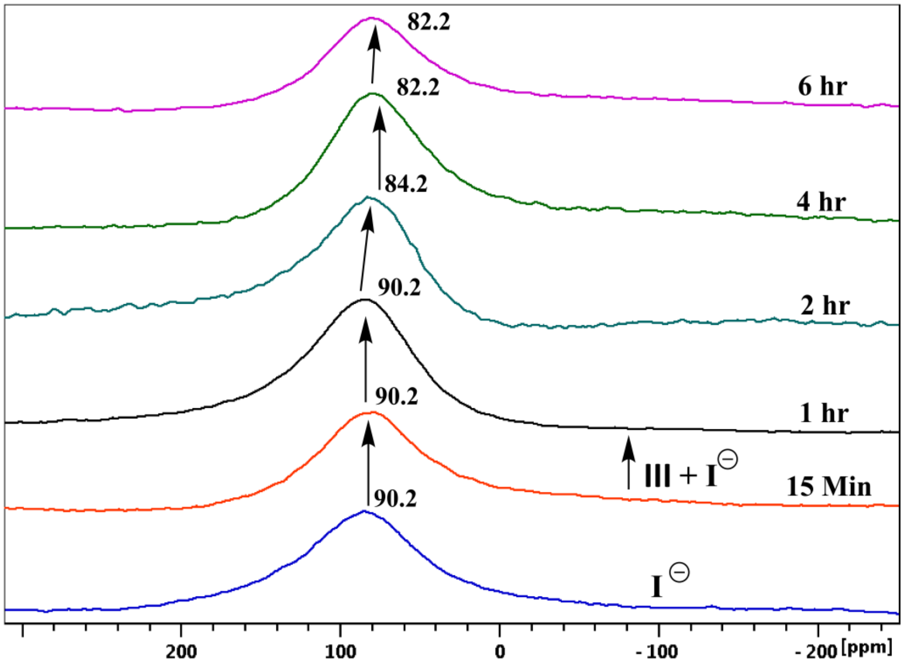

Figure S13. Time-dependent ^{127}I NMR spectra (500 MHz) in $CDCl_3$: CD_3CN at 25˚C showing ^{127}I peaks of Tetrabutylammonium Iodide absence, as well as in the presence, of Ionophore **III**.

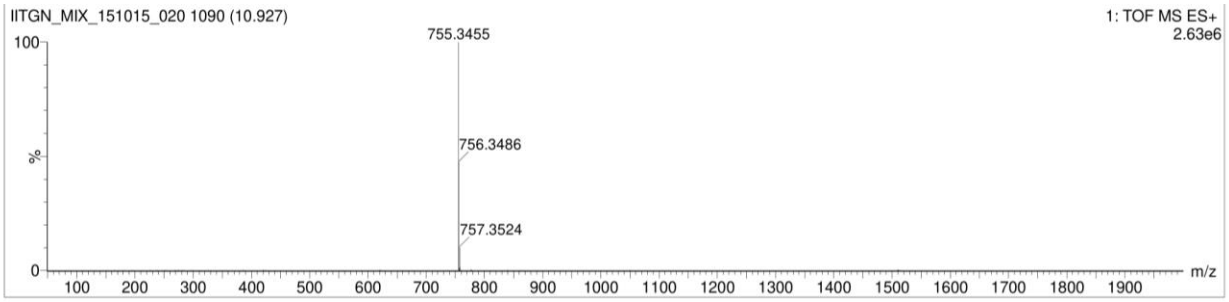

Figure S14. Mass Spectra of ionophore **I**.

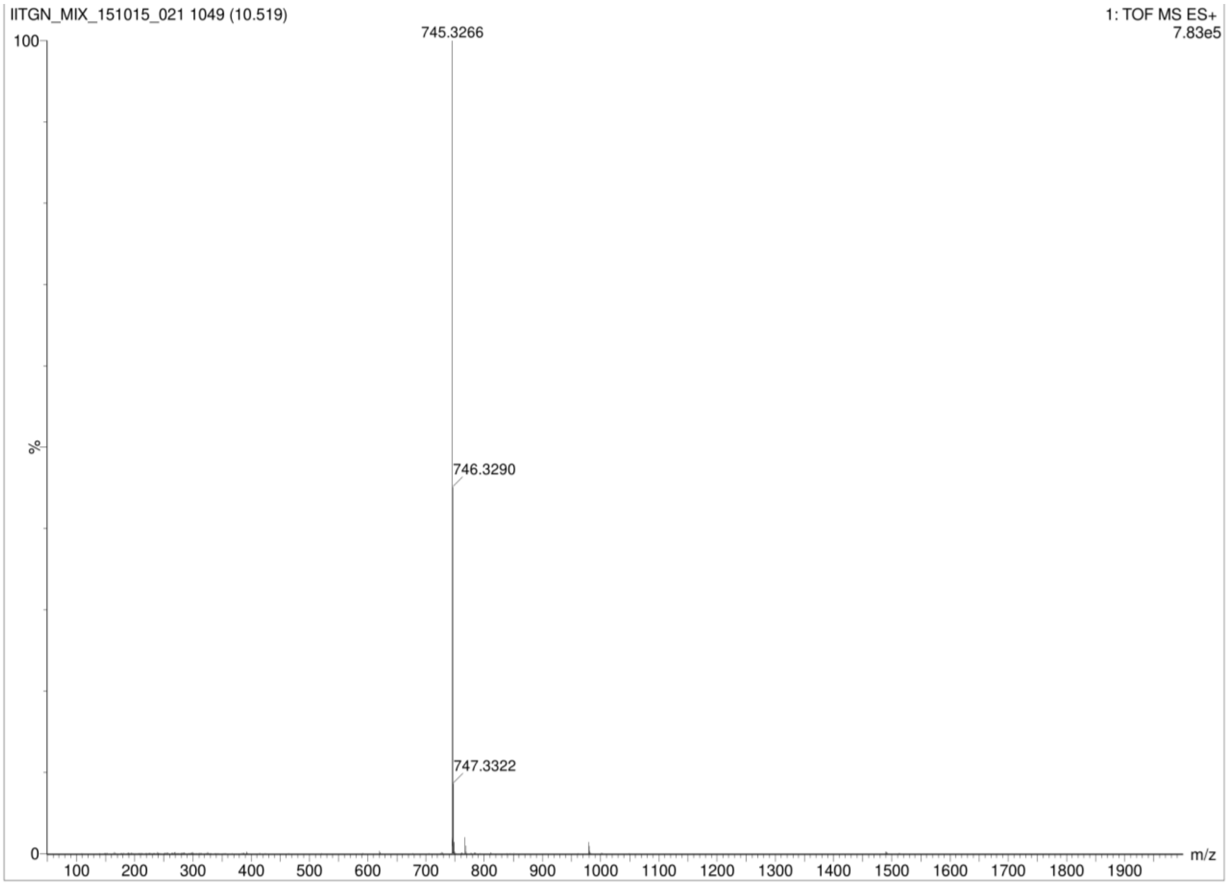

Figure S15. Mass Spectra of ionophore **II**.

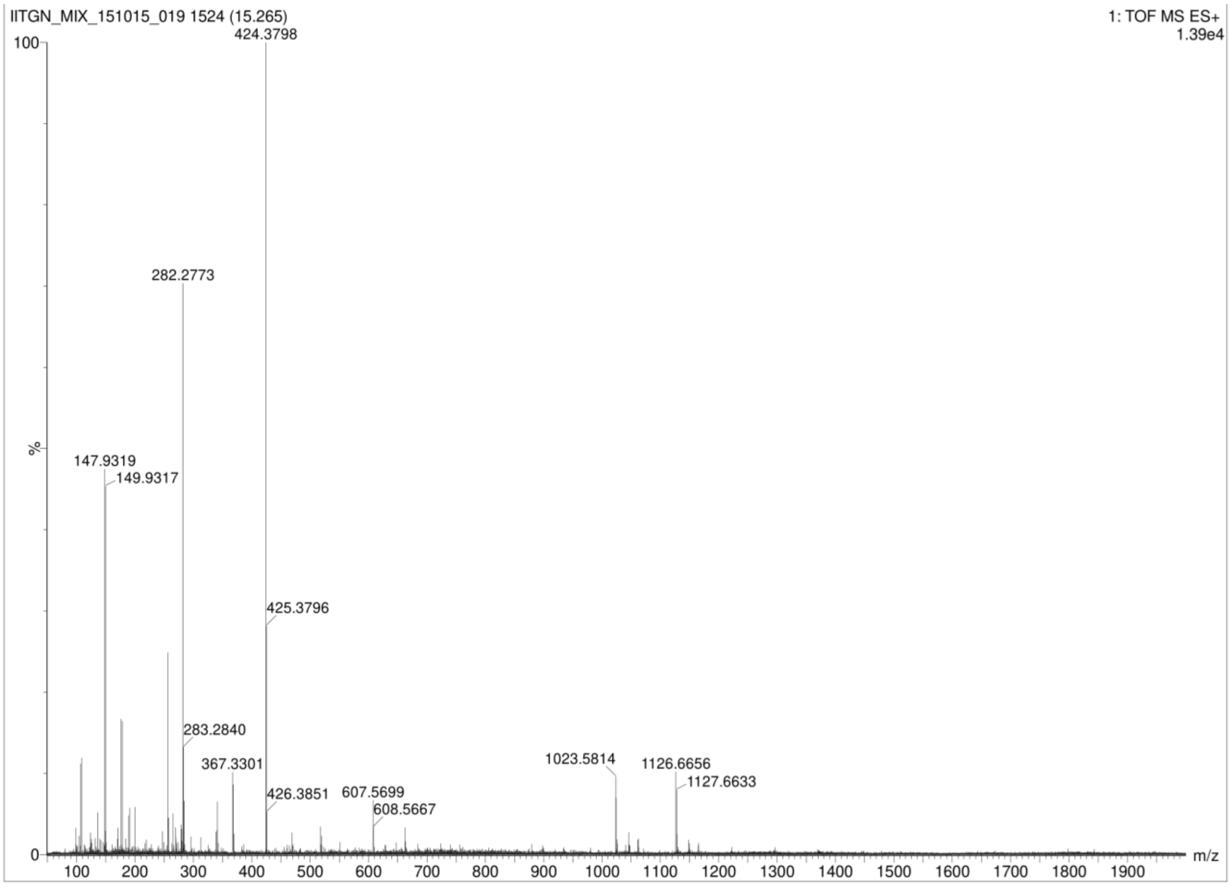

Figure S16. Mass Spectra of ionophore **III**

Table S1. X-ray crystal structure data of Ionophore I

Ionophore	I
Empirical Formula	$C_{46}H_{47}Cl_3N_4O_{10}$
Formula mass	922.23
Crystal color	Colorless
Crystal size	0.21x0.12x0.11
Temperature (K)	298 (2)
Crystal system	Monoclinic
Space group	P 21/c
Unit cell dimensions	a = 14.3406(8) Å, b = 17.5842(11) Å, c = 19.2583(11) Å
Cell Angles	α = 90.00°, β = 105.478(3)°, γ = 90.00°

Cell Volume	4680.2(5) Å3
Z	4
Calculated Density	1.309 g/cm3
Abs. Coeff. (mm-1)	0.256
F(000)	1928
Reflection no. total	8217
Reflection got	5666
No. of parameter	570
S (GOF)on F2	1.925
Final R1 WR2(l>21)weighted R1, WR2 (all data)	0.3244 , 0.2791
R_{all}	0.1390
CCDC Number	1506051

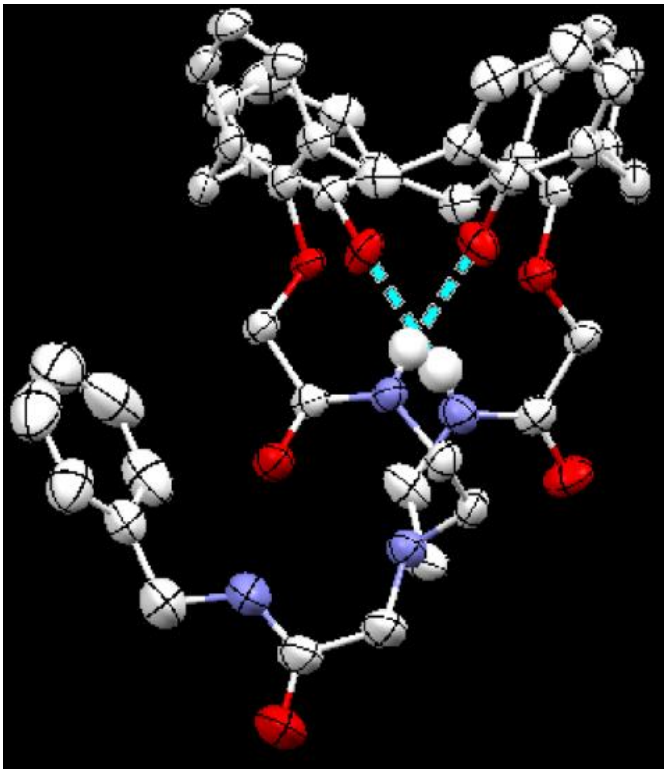

Figure S17. Single Crystal X-ray Structure of ionophore I (50% probability)

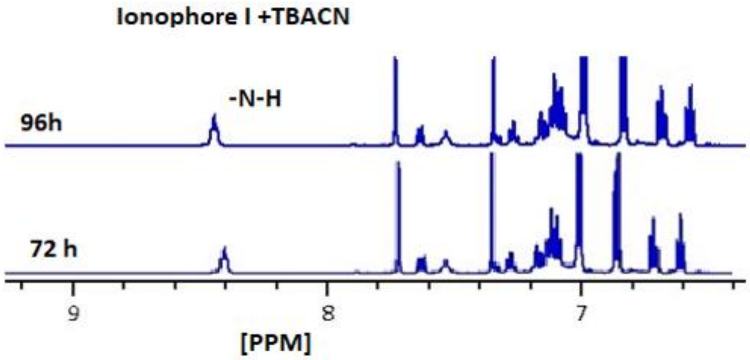

Figure S18: Time dependent NMR when color is reappearing after 72 hrs.
